# Analysis of influenza A, influenza B and *Mycoplasma pneumoniae* infection in children hospitalized with acute respiratory tract infection in Xi’an from 2021 to 2023

**DOI:** 10.3389/fpubh.2026.1762939

**Published:** 2026-03-06

**Authors:** Haijin Zhang, Guihua Zhuang, Bingtong Zhao, Zengguo Wang

**Affiliations:** 1 Department of Epidemiology and Biostatistics, School of Public Health, Xi'an Jiao Tong University Health Science Center, Xi'an, Shaanxi, China; 2 Department of Infection Control, Xi’an Children’s Hospital, Xi’an, Shaanxi, China; 3 Laboratory Department, Xi’an Children’s Hospital, Xi’an, Shaanxi, China

**Keywords:** acute respiratory tract infection, influenza A/B, *Mycoplasma pneumoniae*, pediatric respiratory infection, Xi’an

## Abstract

**Objective:**

To evaluate the occurrence of three pathogens, influenza virus A (FluA), influenza virus B (FluB) and *Mycoplasma pneumoniae* (Mp) in children hospitalized with acute respiratory tract infection (ARTI) between 2021 and 2023.

**Methods:**

FluA, FluB and Mp were detected by antigen/antibody kit in specimens from 29,014 children with ARTI admitted to Xi’an Children’s Hospital.

**Results:**

The total pathogen detection rate was 43.79% (12,707/29014) with 30.14% (8,745/29014) Mp-positive cases, 7.08% (55/29014) FluA-positive and 6.57% (1907/29014) FluB-positive. Total detection rates were 28.05% (2,472/8813) in 2021, 29.38% (2,550/8680) in 2022 and 66.70% (7,685/11521) in 2023. Co-infection rates with two or more pathogens increased over the period of interest from 0.75% (66/8813) in 2021, 0.93% (81/8680) in 2022 to 2.38% (274/11521) in 2023 (*χ*^2^ = 115.931, *p* < 0.001). FluA detection also increased and was present in 0.53% cases in 2021, 6.01% in 2022 and 12.90% in 2023, reaching a peak in February 2023 of 17.83% and March 2023 of 64.42%. FluB detection rates were more stable at 5.25% in 2021, 7.67% in 2022 and 6.75% in 2023, reaching a peak of 22.17% in December 2023. Mp detection was higher in 2023 at 47.05% with a peak of 82.21% in October 2023 than in 2021 (15.89%) or 2022 (15.69%). More FluA-positive cases were seen in the >12 age groups than in younger children and FluB detection also increased with age. Mp infection was detected in the >4 age group.

**Conclusion:**

The pathogen spectrum and epidemiology of ARTI changed between 2021 and 2023. Age-specific bandings of infection were seen in the cohort of children. Outbreaks of FluA, FluB and Mp influence the pathology of ARTI.

## Introduction

Acute respiratory tract infection (ARTI) is a common clinical manifestation of pediatric infections and a leading cause of death in children under 5 years of age. Influenza virus A (FluA), influenza virus B (FluB) and *Mycoplasma pneumoniae* (Mp) are the most prevalent etiological agents (1–3). These pathogens are considered to undergo seasonal variations due to fluctuations in temperature, humidity and population movement. Respiratory syncytial virus (RSV) and influenza virus typically peak during the winter months in temperate regions with enteroviruses circulating more widely in summer and early autumn. Infection patterns are also thought to differ across pediatric age groups. Immune maturation, previous exposure, maternal antibody persistence and age-related differences in social behavior and host receptor expression all contribute to distinct epidemiological profiles for neonates, infants, young children and adolescents. Interactions of seasonal dynamics and age-specific susceptibility are likely to have a large impact on incidence. Greater understanding of these factors is necessary to inform targeted surveillance, optimize immunization schedules and guide clinical management in pediatric populations. The aim of the current study was to analyze the ARTI pathogen spectrum, temporal variation and epidemiological characteristics in pediatric patients admitted to hospital in Xi’an between 2021 and 2023. The influence of pediatric patient age was scrutinized to assess age-related variations in infection pattern and the impact of post-pandemic infection control analyzed. The characterization of infection patterns is intended to inform clinical treatment of pediatric respiratory infections in the region and to increase the level of preparedness for admission.

## Methods

### Clinical data

A total of 29,014 pediatric inpatients, 16,762 males and 12,252 females, age range 1 month to 15 years, diagnosed with ARTI at Xi’an Children’s Hospital between January 1 2021 and December 31 2023 were enrolled. Clinical diagnoses were in accordance with the criteria outlined in the Ninth Edition of Pediatrics. Exclusion criteria were neonates, presence of congenital genetic disorders, immunodeficiency diseases, malignant tumors or prior treatment with radiotherapy, chemotherapy or immunosuppressive therapy. Experiments were performed in alignment with the Declaration of Helsinki (amended 2023) and written informed consent was given by patients’ guardians. Ethical approval was granted by the ethics review committee of Xi’an Children’s Hospital.

### Research methods

#### Sample collection

Throat swab specimens: A tongue depressor was used to immobilize the tongue and a sampling swab passed over the base of the tongue to the posterior pharyngeal wall and tonsillar crypts/lateral walls, 3–5 times. Samples were placed in 3 mL cell preservation solution and stored at −20 °C.

Serum samples: Venous blood samples were drawn, centrifuged at 6000 rpm for 10s and the supernatant stored at −20 °C.

#### Pathogen detection

FluA and FluB were detected by colloidal gold antigen detection kits (Abbott Biologicals Co., Ltd.). Detection of influenza A and B virus antigens was performed using a gold immunochromatographic assay designed for the qualitative detection of influenza A and B viral antigens in human nasopharyngeal aspirates, nasal swabs and throat swabs, based on the principle of sandwich immunocomplex formation. Samples were mixed with a lysis buffer containing surfactants in a TRIS-based solution and antigens migrated chromatographically on the test strip with binding of influenza A or B antigens to colloidal gold-labeled anti-influenza antibodies and of the resulting complexes to immobilized monoclonal antibodies, producing visible purple-red bands. A control of hapten-specific polyclonal antibody was included. Samples of nasal or throat mucosa were collected by sterile swabs, immersed in lysis buffer for approximately 10 s to elute the antigens and results produced by test device after 15 min. Anti-Mp-IgM antibodies were detected by colloidal gold kit (Zhuhai Livzon Diagnostic Inc.). *Mycoplasma pneumoniae* IgM antibodies were detected by gold immunochromatographic assay in which the conjugate pad contained colloidal gold-labeled anti-human IgM (*μ* chain) antibodies with nitrocellulose membrane coated with purified *M. pneumoniae* protein in the test line and goat anti-mouse antibody in the control line. Serum (non-hemolyzed, non-lipemic and non-icteric, stored at 2–8 °C for short-term use or at −20 °C for long-term storage with no more than three freeze–thaw cycles), plasma or anticoagulated whole blood (stored at 2–8 °C and tested within 7 days without freezing or vigorous shaking to prevent hemolysis) samples were examined. Samples were equilibrated to room temperature, applied to the test card and analyzed by GS1201 Colloidal Gold Reader, in accordance with the manufacturer’s standardized protocols. FluA, FluB and Mp nucleic acids in throat swabs samples were identified by RT-PCR. Samples were vortexed for 10s, aspirated and analyzed along with 2 μL internal control.

### Statistical analysis

Statistical analyses were performed using SPSS 19.0 software. Numerical data, such as pathogen detection rates, are expressed as n (%). Comparisons of pathogen detection rates across different age groups, seasons, and years were conducted using Pearson’s Chi-square test or Fisher’s exact test, as appropriate. A *p* value < 0.05 was considered to indicate statistical significance.

## Results

### Baseline characteristics

A cohort of 29,014 pediatric patients diagnosed with ARTI included 16,762 (57.8%) males and 12,252 (42.2%) females with ages ranging from 1 month to 15 years. The total detection rate for all three pathogens was 43.79% (12,707/29,014). Annual total pathogen detection rates were 28.05% (2,472/8,813) in 2021, 29.38% (2,550/8,680) in 2022 and 66.70% (7,685/11,521) in 2023. The highest proportion of ARTI cases occurred in children aged 1–3 years, accounting for 38.33% of the total cohort. Seasonal variation was observed with higher ARTI incidence during the Autumn. Mp was the most prevalent pathogen and was detected in 30.14% (8,745/29,014) cases, followed by FluA in 7.08% (2,055/29,014) and FluB in 6.57% (1,907/29,014). Analysis of pathogen detection by year showed rates of 0.53% (47/8,813) FluA, 5.25% (463/8,813) FluB and 22.26% (1,962/8,813) Mp in 2021; 6.01% (522/8,680) FluA, 7.67% (666/8,680) FluB and 15.69% (1,362/8,680) Mp in 2022; 12.90% (1,486/11,521) FluA, 6.75% (778/11,521) FluB and 47.05% (5,421/11,521) Mp in 2023 (Table 1).

**Table 1:** Demographic characteristics and pathogen detection in children hospitalized with ARTI from 2021 to 2023.

Variable and category	2021 (*N* = 8,813)		2022 (*N* = 8,680)		2023 (*N* = 11,521)		Total (*N* = 29,014)	
	2021 *N*	2021 %	2022 *N*	2022 %	2023 *N*	2023 %	Total *N*	Total %
Gender								
Male	5,214	59.16	5,075	58.47	6,473	56.18	16,762	57.77
Female	3,599	40.84	3,605	41.53	5,048	43.82	12,252	42.23
Age								
1–6 months	1,582	17.95	1,188	13.69	1,256	10.90	4,026	13.88
7–12 months	986	11.19	814	9.38	729	6.33	2,529	8.72
1–3 years	4,000	45.39	3,479	40.08	3,642	31.61	11,121	38.33
4–6 years	1,540	17.47	1981	22.82	2,990	25.95	6,511	22.44
7–12 years	655	7.43	1,147	13.21	2,780	24.13	4,582	15.79
>12 years	50	0.57	71	0.82	124	1.08	245	0.84
Season								
Spring	2,118	24.03	2015	23.21	3,125	27.12	7,258	25.02
Summer	2,201	24.97	2,596	29.91	2,252	19.55	7,049	24.30
Autumn	2,157	24.48	2,552	29.40	3,398	29.49	8,107	27.94
Winter	2,337	26.52	1,517	17.48	2,746	23.83	6,600	22.75
Pathogen								
Flu A	47	0.53	522	6.01	1,486	12.90	2055	7.08
Flu B	463	5.25	666	7.67	778	6.75	1907	6.57
Mp	1962	22.26	1,362	15.69	5,421	47.05	8,745	30.14

### Patterns of ARTI pathogen infection

Detection rates in single-pathogen infections followed the pattern of Mp > FluB > FluA for 2021 and 2022. Mp remained the most frequently detected pathogen in 2023 but FluA rates exceeded those of FluB relative to 2021 and 2022 (χ^2^ = 1063.879, *p* < 0.001). Mixed-pathogen infections were observed in 0.75% (66/8,813) cases in 2021, 0.93% (81/8,680) in 2022 and 2.38% (274/11,521) in 2023, indicating a significant year-on-year increase (χ^2^ = 115.931, *p* < 0.001). No cases of triple-pathogen co-infection were detected in 2021. One such case was identified in 2022 and five in 2023 (Table 2).

**Table 2:** Presence of single, double and triple pathogen infections in children hospitalized with ARTI from 2021 to 2023.

Epidemiological pattern	2021 (*N* = 8,813)		2022 (*N* = 8,680)		2023 (*N* = 11,521)		*χ* ^2^	*p*
	*N*	%	*N*	%	*N*	%		
Single infection								
FluA	40	0.45	503	5.79	1,362	11.82	1063.879	<0.001
FluB	402	4.56	602	6.94	608	5.28	49.797	<0.001
Mp	1898	21.54	1,285	14.80	5,162	44.81	2497.126	<0.001
Double infection								
FluA + FluB	2	0.02	3	0.03	15	0.13	10.501	0.005
FluA + Mp	5	0.06	16	0.18	104	0.90	100.847	<0.001
FluB+Mp	59	0.67	61	0.70	150	1.30	28.641	<0.001
Triple infection								
FluA + FluB + Mp	0	0.00	1	0.01	5	0.04	–	0.082*

*Fisher’s exact probability test.

### Monthly detection rates

FluA detection rates remained <1% throughout 2021 but began to rise in July 2022, reaching a peak of 15.99% in November (Table 3, *p* < 0.001). Sporadic cases were detected in January 2023 and a marked increase occurred between February (17.83%) and March (64.42%) (Table 3, *p* < 0.001) with a rapid decrease by May. A second, lower peak emerged in November (8.51%) and December (18.98%, Table 3). Rates of FluB detection were low, at <5%, for most of 2023 but rose in November giving the highest monthly detection rate in December 2023 at 22.17%, indicating a pronounced winter peak (Table 4, *p* < 0.001). FluB detection was higher during the summer and autumn of 2022, compared with these seasonal periods in 2021 and 2023 (Table 4, *p* < 0.001). More cases of Mp infection were detected in 2023 than in 2021 or 2022, although no seasonal pattern was observed. Detection rates were lower from January to June 2023 than in the corresponding months of the previous 2 years but a marked upward trend emerged from June 2023, reaching a peak in the October of 82.21%, which then declined (Table 5, *p* < 0.001).

**Table 3:** FluA detection rate, 2021 to 2023.

Month	2021			2022			2023			*χ* ^2^	*p*
	Number of tests	Number of positives	Positivity rate (%)	Number of tests	Number of positives	Positivity rate (%)	Number of tests	Number of positives	Positivity rate (%)		
January	1,102	3	0.27	335	2	0.60	422	3	0.71	–	0.332*
February	512	6	1.17	318	1	0.31	443	79	17.83	132.580	<0.001
March	490	0	0.00	562	4	0.71	1,186	764	64.42	1014.337	<0.001
April	728	2	0.27	661	1	0.15	924	129	13.96	198.128	<0.001
May	900	1	0.11	792	1	0.13	1,015	12	1.18	–	0.001*
June	867	2	0.23	906	1	0.11	849	6	0.71	–	0.10*
July	783	4	0.51	884	13	1.47	711	4	0.56	5.56	0.062
August	551	12	2.18	806	59	7.32	692	5	0.72	50.325	<0.001
September	429	5	1.17	840	134	15.95	866	4	0.46	190.021	<0.001
October	814	5	0.61	855	109	12.75	1,169	7	0.60	215.828	<0.001
November	914	3	0.33	857	137	15.99	1,363	116	8.51	144.932	<0.001
December	723	4	0.55	864	60	6.94	1881	357	18.98	195.364	<0.001
Total	8,813	47	0.53	8,680	522	6.01	11,521	1,486	12.90	1181.539	<0.001

**Table 4:** FluB detection rate, 2021 to 2023.

Months	2021			2022			2023			*χ* ^2^	*p*
	Number of tests	Number of positives	Positivity rate (%)	Number of tests	Number of positives	Positivity rate (%)	Number of tests	Number of positives	Positivity rate (%)		
January	1,102	28	2.54	335	44	13.13	422	53	12.56	75.605	<0.001
February	512	30	5.86	318	35	11.01	443	29	6.55	8.296	0.016
March	490	22	4.49	562	37	6.58	1,186	46	3.88	6.297	0.043
April	728	53	7.28	661	17	2.57	924	35	3.79	19.729	<0.001
May	900	58	6.44	792	15	1.89	1,015	19	1.87	38.099	<0.001
June	867	31	3.58	906	34	3.75	849	29	3.42	0.144	0.930
July	783	26	3.32	884	38	4.30	711	12	1.69	8.741	0.013
August	551	15	2.72	806	78	9.68	692	14	2.02	53.590	<0.001
September	429	14	3.26	840	105	12.50	866	26	3.00	71.319	<0.001
October	814	44	5.41	855	105	12.28	1,169	9	0.77	124.513	<0.001
November	914	58	6.35	857	120	14.00	1,363	89	6.53	45.522	<0.001
December	723	84	11.62	864	38	4.40	1881	417	22.17	153.153	<0.001
Total	8,813	463	5.25	8,680	666	7.67	11,521	778	6.75	42.689	<0.001

**Table 5:** Mp detection rate, 2021 to 2023.

Months	2021			2022			2023			*χ* ^2^	*p*
	Number of tests	Number of positives	Positivity rate (%)	Number of tests	Number of positives	Positivity rate (%)	Number of tests	Number of positives	Positivity rate (%)		
January	1,102	234	21.23	335	67	20.00	422	85	20.14	0.366	0.833
February	512	90	17.58	318	59	18.55	443	52	11.74	8.527	0.014
March	490	127	25.92	562	76	13.52	1,186	72	6.07	127.795	<0.001
April	728	163	22.39	661	89	13.46	924	77	8.33	66.382	<0.001
May	900	236	26.22	792	117	14.77	1,015	94	9.26	102.002	<0.001
June	867	192	22.15	906	99	10.93	849	226	26.62	73.003	<0.001
July	783	169	21.58	884	91	10.29	711	305	42.90	234.289	<0.001
August	551	106	19.24	806	134	16.63	692	518	74.86	643.587	<0.001
September	429	93	21.68	840	131	15.60	866	658	75.98	726.670	<0.001
October	814	156	19.16	855	123	14.39	1,169	961	82.21	1202.377	<0.001
November	914	233	25.49	857	133	15.52	1,363	1,078	79.09	1075.841	<0.001
December	723	163	22.54	864	243	28.13	1881	1,295	68.85	649.589	<0.001
Total	12,348	1962	15.89	8,680	1,362	15.69	11,521	5,421	47.05	3698.357	<0.001

### Age group analysis

Separation of pediatric ARTI patients into six age groups, 1–6 months, 7–12 months, 1–3 years, 4–6 years, 7–12 years and >12 years, showed that FluA detection rates were low in all groups in 2021. The highest 2022 FluA detection rates were seen in the 4–6 year group at 11.26% (223/1981) and rates were higher in all age groups in 2023 compared with 2021 and 2022, with the highest rate among >12 s (22.58%, 28/124), followed by 1–3 and 4–6 years.

FluB detection increased with age. Rates were low in children under 1 year, ranging from 0.57 to 2.07%, but higher for the >12 group, at 22.00% in 2021, 36.62% in 2022 and 27.42% in 2023. Mp was detected in all age groups with the highest rates in school-aged children of 7–12 and >12 years. The 7–12 year group showed a 65.65% Mp detection rate in 2021, rising sharply to 85.18% in 2023. Mp detection rates were lower in all age groups in 2022 than in 2021 and 2023 (Table 6).

**Table 6:** Detection of FluA, FluB and Mp in different age groups of children hospitalized with ARTI from 2021 to 2023.

Age group	2021			2022			2023			*χ* ^2^	*p*
	Number of tests	Number of positives	Positivity rate (%)	Number of tests	Number of positives	Positivity rate (%)	Number of tests	Number of positives	Positivity rate (%)		
FluA											
1–6 months	1,582	2	0.13	1,188	20	1.68	1,256	95	7.56	146.176	<0.001
7–12 months	986	2	0.20	814	16	1.97	729	88	12.07	161.826	<0.001
1–3 years	4,000	28	0.70	3,479	192	5.52	3,642	542	14.88	614.826	<0.001
4–6 years	1,540	5	0.32	1981	223	11.26	2,990	431	14.41	225.868	<0.001
7–12 years	655	7	1.07	1,147	70	6.10	2,780	302	10.86	76.510	<0.001
>12 years	50	3	6.00	71	1	1.41	124	28	22.58	20.581	<0.001
FluB											
1–6 months	1,582	9	0.57	1,188	9	0.76	1,256	26	2.07	16.349	<0.001
7–12 months	986	10	1.01	814	13	1.60	729	16	2.19	3.871	0.144
1–3 years	4,000	185	4.63	3,479	236	6.78	3,642	215	5.90	16.421	<0.001
4–6 years	1,540	132	8.57	1981	210	10.60	2,990	229	7.66	12.988	0.002
7–12 years	655	116	17.71	1,147	172	15.00	2,780	258	9.28	49.708	<0.001
>12 years	50	11	22.00	71	26	36.62	124	34	27.42	6.214	0.045
Mp											
1–6 months	1,582	30	1.90	1,188	15	1.26	1,256	72	5.73	52.648	<0.001
7–12 months	986	47	4.77	814	28	3.44	729	99	13.58	72.991	<0.001
1–3 years	4,000	840	21.00	3,479	401	11.53	3,642	1,016	27.90	296.702	<0.001
4–6 years	1,540	569	36.95	1981	420	21.20	2,990	1801	60.23	770.008	<0.001
7–12 years	655	430	65.65	1,147	453	39.49	2,780	2,368	85.18	832.707	<0.001
>12 years	50	46	92.00	71	45	63.38	124	65	52.42	24.138	<0.001

Children aged 1–3 years accounted for the highest proportion of FluA infections in 2021 at 59.7%. This proportion decreased to 36.78% in 2022 and 36.47% in 2023. The proportion of FluA-positive cases in the 7–12 year group increased markedly in 2023, reaching a peak of 20.32%. By contrast, FluB-infected ARTI cases in the 1–3 year group declined from 39.96% in 2021 to 27.63% in 2023. Proportions in the 4–6 year group remained relatively constant from 2021 to 2023, ranging between 28.51 and 31.53%, while those in the 7–12 year group increased to 33.16% in 2023. Mp infections decreased from 42.81% in 2021 to 18.74% in 2023 in those aged 1–3 years but a consistent increase was seen in the 7–12 year group from 21.92% in 2021, 33.26% in 2022 to 43.68% in 2023. Distributions in other age groups remained relatively stable throughout the study period (Table 7).

**Table 7:** Changes in FluA, FluB and Mp infection rates by calendar year and age grouping from 2021 to 2023.

Age group	2021		2022		2023	
	*N*	%	*N*	%	*N*	%
FluA						
1–6 months	2	4.26	20	3.83	95	6.39
7–12 months	2	4.26	16	3.07	88	5.92
1–3 years	28	59.57	192	36.78	542	36.47
4–6 years	5	10.64	223	42.72	431	29.00
7–12 years	7	14.89	70	13.41	302	20.32
>12 years	3	6.38	1	0.19	28	1.88
Total	47	100.00	522	100.00	1,486	100.00
FluB						
1–6 months	9	1.94	9	1.35	26	3.34
7–12 months	10	2.16	13	1.95	16	2.06
1–3 years	185	39.96	236	35.44	215	27.63
4–6 years	132	28.51	210	31.53	229	29.43
7–12 years	116	25.05	172	25.83	258	33.16
>12 years	11	2.38	26	3.90	34	4.37
Total	463	100.00	666	100.00	778	100.00
Mp						
1–6 months	30	1.53	15	1.10	72	1.33
7–12 months	47	2.40	28	2.06	99	1.83
1–3 years	840	42.81	401	29.44	1,016	18.74
4–6 years	569	29.00	420	30.84	1801	33.22
7–12 years	430	21.92	453	33.26	2,368	43.68
>12 years	46	2.34	45	3.30	65	1.20
Total	1962	100.00	1,362	100.00	5,421	100.00

## Discussion

The anatomical structure of the respiratory tract differs in children from that of adults and pediatric airway immunological functions are relatively underdeveloped. As a result, children are more susceptible to acute respiratory tract infections (ARTIs) than adults. Many pathogens may cause pediatric ARTI with FluA and FluB being the most common etiological agents. Mp is more atypical but is also a major cause of ARTI (4–6). The epidemiology and trends of respiratory pathogens in pediatric patients from 2021 to 2023 were analyzed in the current study with the aim of informing the diagnosis and clinical management of pediatric ARTI in the Xi’an region.

Some significant shifts in pathogen prevalence were recorded and these were likely to have been influenced by epidemic characteristics. Similar rebounds have been reported elsewhere. The United States saw its 2022–2023 influenza season make an unusually early return to pre-COVID-19 levels (7) and Europe experienced an early and intense flu epidemic in winter 2022–2023, marking a return of influenza to near pre-pandemic levels (8). East Asia also saw atypical patterns of high influenza incidence in 2023 (9). Influenza is known to vary with the season, typically peaking in winter and spring, with outbreaks often lasting 3–4 weeks before subsiding naturally. Influenza has characteristics of high morbidity but relatively low mortality rates. FluA is an enveloped RNA virus which belongs to the Orthomyxoviridae family. It has a 1–3 day incubation period, after which fever, cough, sore throat, myalgia and headache are frequent symptoms (10–13). FluA showed two epidemic peaks in February and November 2023 among children with ARTI with a particularly high detection rate of 64.42% in March. Detection rates recorded for FluA were 0% in 2021 and 0.71% in 2022 for equivalent seasonal periods. A similar trend was shown by surveillance data from the Chinese National Influenza Center with a sharp increase in Week 7 of 2023, peaking at 99.99% in Week 11, and co-circulation of seasonal influenza A(H1N1) pdm09 and A(H3N2) subtypes. FluA-positive rates declined steadily thereafter, returning to pre-epidemic levels by Week 17. This pattern may be interpreted in the context of reduced mask-wearing and increased population density. It has been observed that FluA incidence increased as adherence to the directives concerning social gatherings and protective measures stemming from the COVID-19 pandemic were relaxed (14). Non-pharmaceutical interventions (NPIs) regarding hand hygiene, mask mandates, social distancing, restrictions on mass gatherings and home isolation disrupted transmission of many respiratory pathogens. The resulting reduction in childhood exposure may have decreased children’s immune stimulation, leading to lower baseline immunity and increased susceptibility to FluA (15, 16). Children are acknowledged to be highly susceptible to influenza and herd immunity may have also been reduced due to low uptake of influenza vaccination during the COVID-19 pandemic.

FluB infection typically presents with acute respiratory symptoms, such as fever, cough and rhinorrhea. FluB hemagglutinin and neuraminidase are more stable than those of FluA, showing lower rates of time-dependent mutation. This characteristic may partially explain the reduced transmissibility of FluB (17–19). No significant variation in FluB detection was found between 2021 and 2023, although there were lower detection rates during spring and summer and an upward trend beginning in November, consistent with seasonal influenza virus patterns.

Mp may cause both upper and lower respiratory tract infections and is a major contributor to outbreaks of atypical pneumonia in children. Transmission occurs primarily through respiratory droplets from coughs or sneezes and the incubation period of 2–4 weeks means that infections may persist within communities for several weeks, making children particularly susceptible to infection (20–22). Mp was the most frequently detected pathogen in children of the current cohort with ARTI in 2023 and the infection rate was 47.05%. Mp detection began to increase above pre-pandemic levels in June 2023, peaking at 82.21% in the October. This spike, peaking in early autumn 2023, likely reflects the delayed re-emergence of Mp after years of low circulation during COVID-19. Indeed, a global outbreak of Mp in late 2023 was reported, especially affecting East Asian countries. South Korea, for example, observed a nationwide epidemic of pediatric Mp pneumonia in 2023–24 (23) and European countries, such as Denmark, noted Mp infections reaching epidemic levels in Autumn 2023 (24). The United States saw a similar rise slightly later with pediatric Mp -associated pneumonias surging in 2024 after remaining low in 2020–2023 (25). We believe that several factors may have contributed to this phenomenon. Practices of mask-wearing, hand hygiene and keeping children at home were adopted in response to the epidemic of FluA during March–April 2023, but relaxed in the summer of that year, which may have facilitated Mp spread. In addition, the previous COVID-19 pandemic control measures may have reduced children’s exposure to common respiratory pathogens, such as Mp, leading to lower baseline immunity and increased susceptibility once interventions were lifted (26, 27). There is also no commercially available Mp vaccine, leaving children without vaccine-induced immunity and universally susceptible to infection (4). Mp infections were predominantly concentrated in children aged 4–6 years and older who are likely to attend kindergarten or primary school. The lack of social distancing and high-density group activities may facilitate the transmission of respiratory pathogens in this age grouping. The current findings suggest that preschool and school-aged children are high-risk groups for respiratory pathogen infections and should be prioritized in public health efforts aimed at controlling and preventing spread.

Mp consistently had the highest detection rate among the pathogens analyzed from 2021 to 2023 during the current work. However, FluA detection increased in 2023, making it the second most common pathogen responsible for single-pathogen infections. The presence of mixed infections rose from 0.75% in 2021 to 2.38% in 2023 and the most frequently identified co-infection was FluB + Mp. No consensus has been reached regarding the underlying causes of co-infection (28, 29) and the impact on clinical disease severity remains controversial (30). Chen et al. (31) and Asner et al. (32) have indicated no significant difference in clinical disease severity between viral co-infections and single respiratory infections. The occurrence of mixed infections may be attributed to the relative immaturity of the pediatric immune system which makes patients vulnerable to simultaneous infection by multiple pathogens.

Pathogen detection showed different patterns according to age groupings for the current cohort. The highest detection rates of FluA and FluB in 2022 were observed in children over 3 years, perhaps due to high population density in childcare facilities and schools increasing pathogen exposure and cross-infection. Children over 3 years represent a high-risk group for respiratory infections and should be a key target for influenza prevention and control (33).

Mp detection increased with age grouping between 2021 and 2023, exceeding 40% in children over 7 years, and primarily affecting school-aged children and adolescents. Our hospital is a children’s specialty facility and the proportion of middle school students, aged >12 years, was relatively low in the current cohort. The proportion of Mp-positive cases increased with age in groupings other than the over 12 s. It may be concluded that there is a strong association between pathogen type in ARTI patients and patient age. Differences in infection patterns are likely to be influenced by variations in social contact and pathogen exposure between home-based and school-attending children. Such observations may inform clinical practice by highlighting the need for vigilance and age-specific management to avoid the rapid progression of illness in children.

Our study has several limitations. First, the retrospective study design may introduce biases related to test ordering practices. Second, co-infections of target pathogens were analyzed but detailed evaluation of underlying comorbidities beyond the exclusion criteria was not performed. Third, molecular characterization and antibiotic susceptibility testing for *M. pneumoniae* were not conducted, which limits interpretations of potential resistance patterns. Finally, as this study included only hospitalized children, the findings may not apply to mild cases treated in outpatient settings.

## Conclusion

The spectrum of pathogens causing ARTI in children in Xi’an underwent significant changes from 2021 to 2023. Pathogen transmission dynamics and susceptibility have adapted to changes in public health behavior, social interaction and vaccination practices, leading to shifts in the epidemiological characteristics of ARTIs. Such evolving trends should be tracked by continuous surveillance and monitoring of pathogen profiles. Extra vigilance is warranted to detect the emergence of novel viral variants and the circulation of additional respiratory pathogens. Implications of these findings suggest that clinical management strategies should be adjusted based on patient age and prevailing seasonal trends. We recommend that public health authorities increase surveillance of Mp in schools and kindergartens. Mp vaccines should also be developed to optimize influenza vaccination coverage and mitigate future outbreaks.

## Data Availability

The raw data supporting the conclusions of this article will be made available by the authors, without undue reservation.
