# Correction: Analysis of influenza A, influenza B and *Mycoplasma pneumoniae* infection in children hospitalized with acute respiratory tract infection in Xi'an from 2021 to 2023

**DOI:** 10.3389/fpubh.2026.1828826

**Published:** 2026-03-23

**Authors:** Haijin Zhang, Guihua Zhuang, Bingtong Zhao, Zengguo Wang

**Affiliations:** 1 Department of Epidemiology and Biostatistics, School of Public Health, Xi'an Jiao Tong University Health Science Center, Xi'an, Shaanxi, China; 2 Department of Infection Control, Xi'an Children's Hospital, Xi'an, Shaanxi, China; 3 Laboratory Department, Xi'an Children's Hospital, Xi'an, Shaanxi, China

**Keywords:** acute respiratory tract infection, influenza A/B, *Mycoplasma pneumoniae*, pediatric respiratory infection, Xi'an

Affiliation Department of Infection Control, Xi'an Children's Hospital, Xi'an, Shaanxi, China was erroneously given as Department of Disease, Xi'an Children's Hospital, Xi'an, Shaanxi, China.

Affiliation “Department of Epidemiology and Biostatistics, School of Public Health, Xi'an Jiao Tong University Health Science Center, Xi'an, Shaanxi, China” was omitted for author Guihua Zhuang. This affiliation has now been added for author Guihua Zhuang.

Author Guihua Zhuang was erroneously assigned to affiliation Department of Disease, Xi'an Children's Hospital, Xi'an, Shaanxi, China. This affiliation has now been removed for author Guihua Zhuang.

The original version of this article has been updated.

